# The Philosophy of Cataplasms

**Published:** 1870-04

**Authors:** 


### The Philosophy of Cataplasms.

The *Journal des Connaissances Medicales* publishes an article, by Dr. Herbert, on a subject which may not be uninteresting to families: viz., cataplasms, those especially which have mustard for their base. The seeds of the black kind, which, in a pulverized state, are used for poultices, owe their proprieties to a liquid, acid, and volatile substance, being nothing but essence of mustard. This, however, does not exist ready formed in the seed; it is generated by a kind of fermentation, caused by the action of an albuminoid body, called myrosine, which plays the part of leaven, on a peculiarly fermentescible compound, mysonate of potash. This transformation which has been called *sinapisic*, can only take place by the intervention of water at a temperature higher than freezing point, and lower than seventy-five deg. centigrade, those being the usual conditions requisite for producing fermentation. This is a circumstance which is not commonly taken into account in practice. The generation of essence of mustard diminishes under temperature ranging between fifty deg. and seventy-five deg. centigrade, and entirely ceases at the latter. Hence, boiling water, or even such that cannot be born by the hand, will spoil both the poultice and the sinapized foot-bath. Again, alcohol, acids, metallic salts, and any other agents having the power of stopping fermentation or retarding it, are detrimental. Besides the two principles mentioned, through whose joint action the essential oil of mustard is produced, the seeds of this plant contain various others, among which there is a fixed and inactive oil, having some of the properties of that of rapeseed, and which may easily be extracted from mustard-powder, either by strong pressure, or, better still, by acting upon it by lixiviation in sulphuret of carbon. When this oil is extracted, what remains is much more powerful, and will, moreover, keep indefinitely. Many years ago, M. Robinet attempted to bring this mustard-flour, deprived of its fixed oil, into general use; but prejudice and routine proved to strong for him; and it was not until this powder was gummed to paper, then cut into squares, and sold in elegant tin boxes, that it came into fashion. But what every family should keep in mind is this, that mustard poultices ought not to be made with hot but lukewarm water.—*The Druggist.—N. Y. Med. Journal.*

